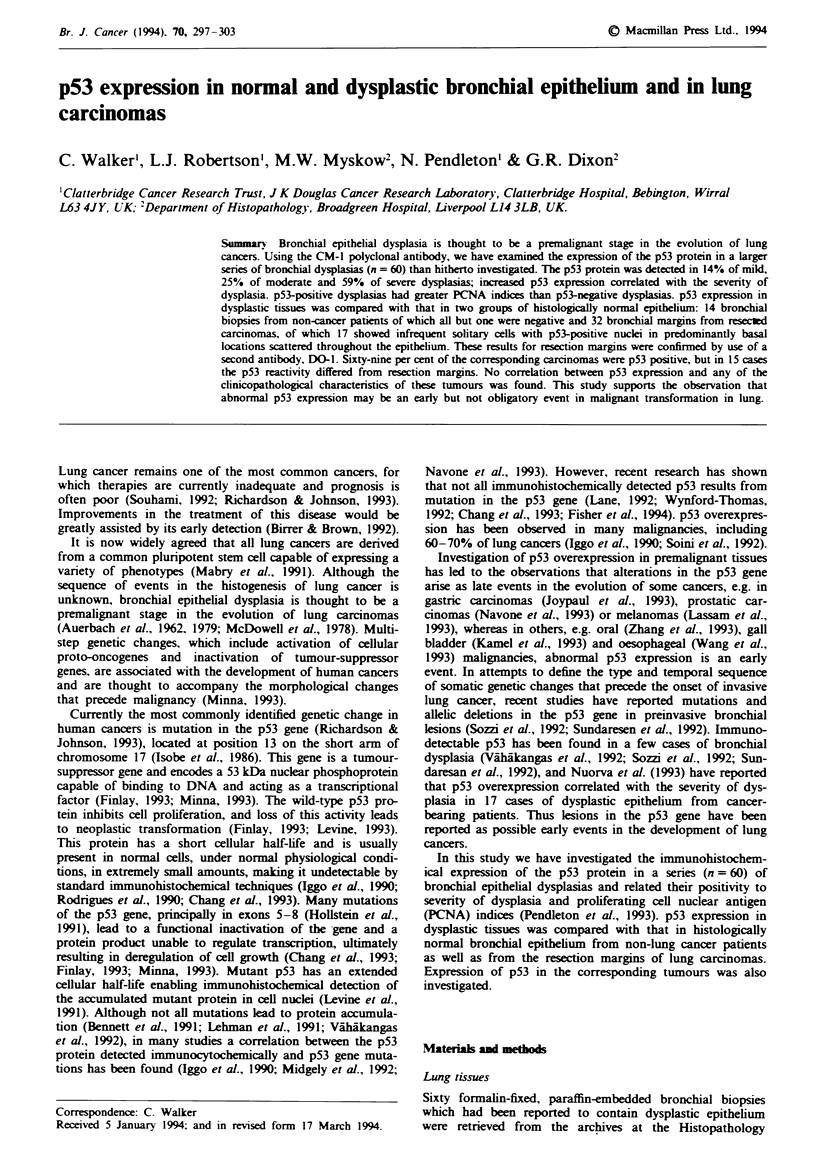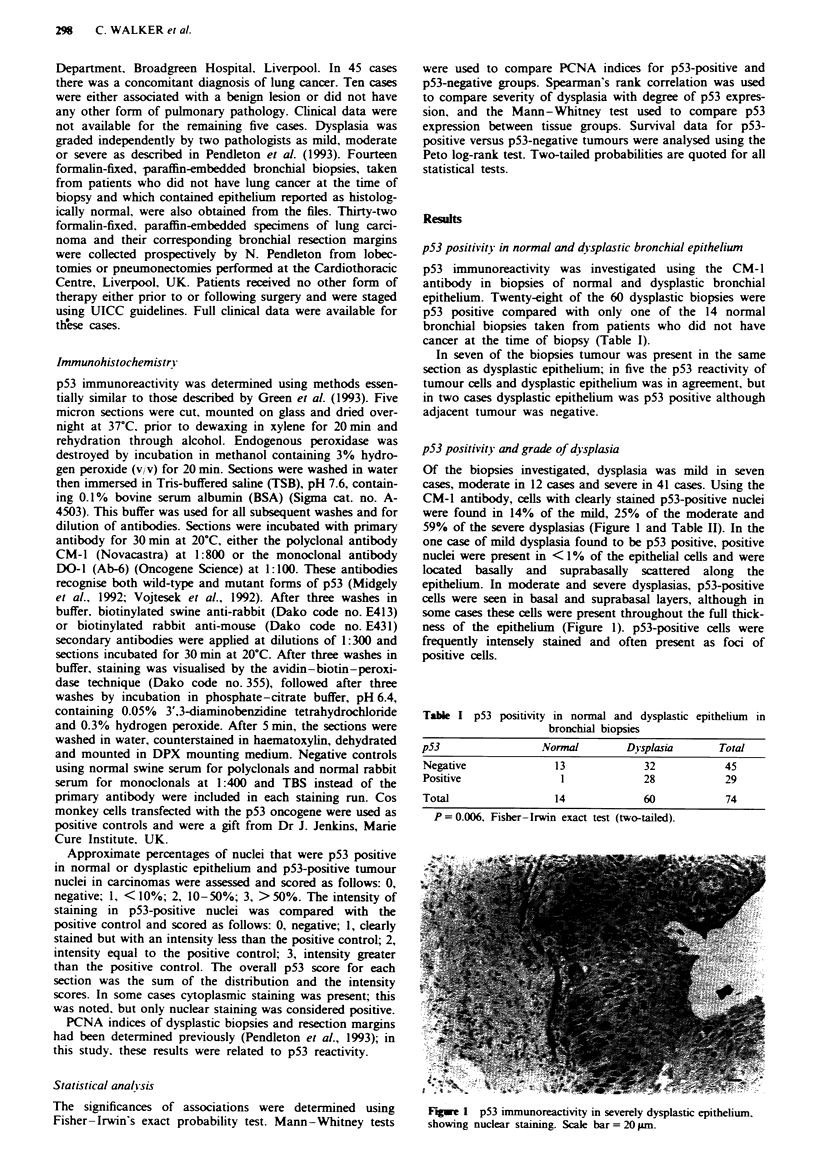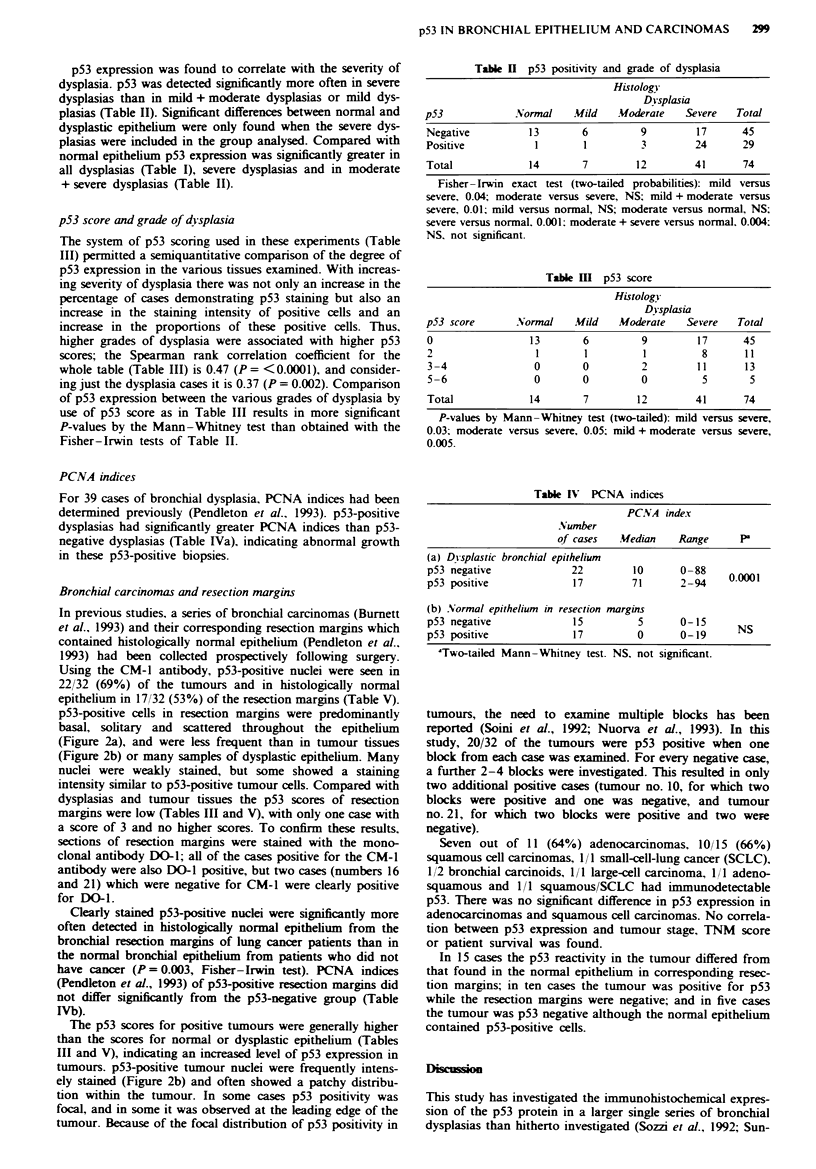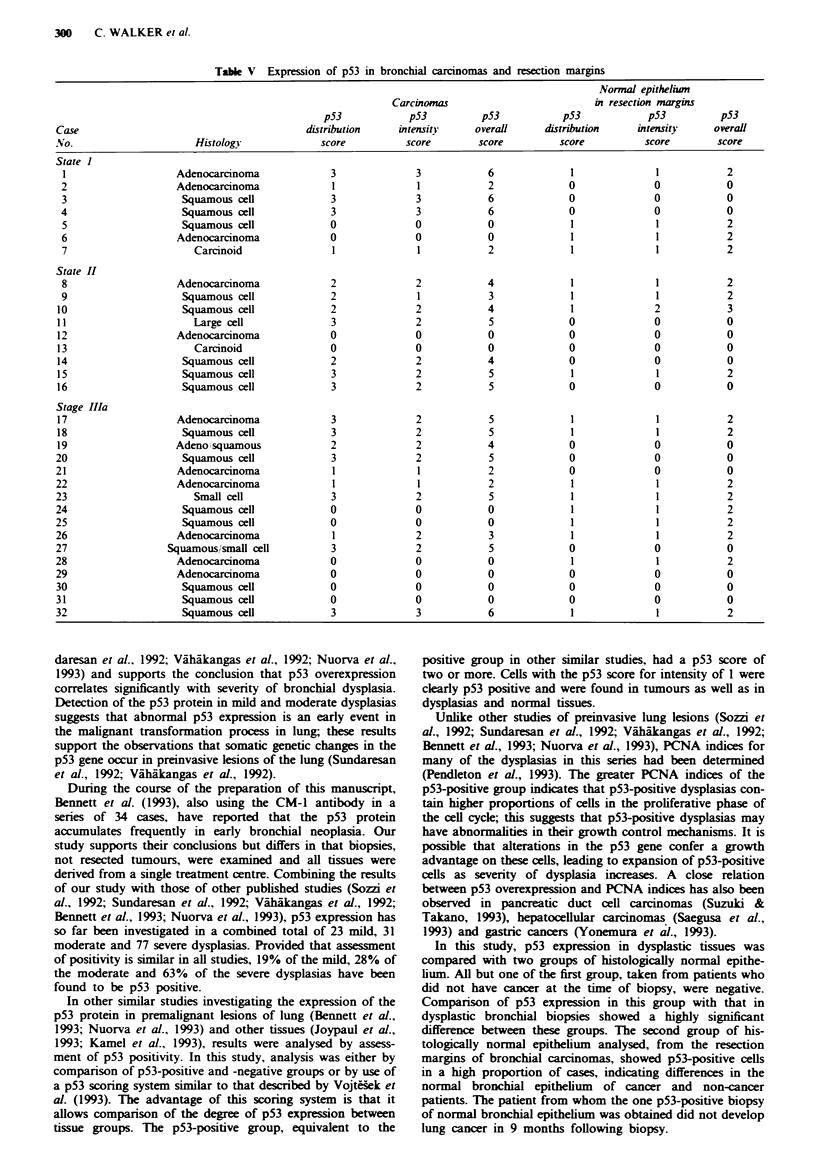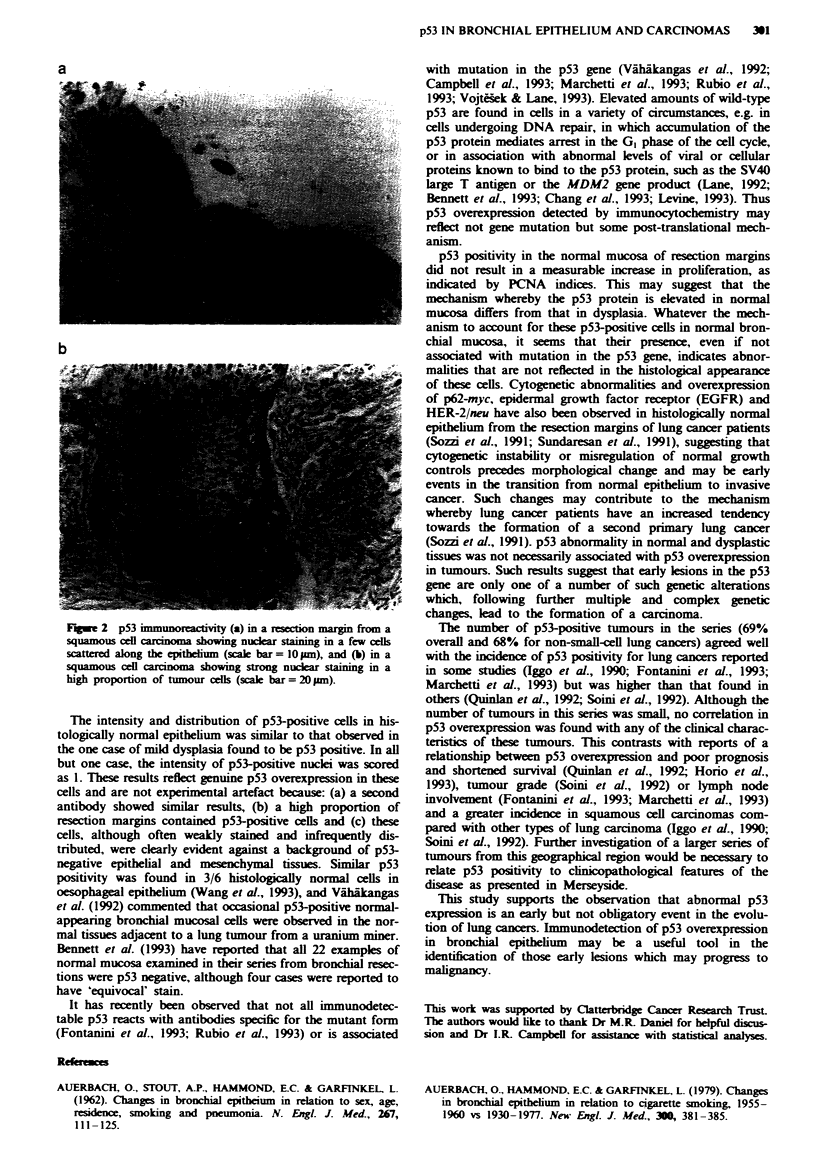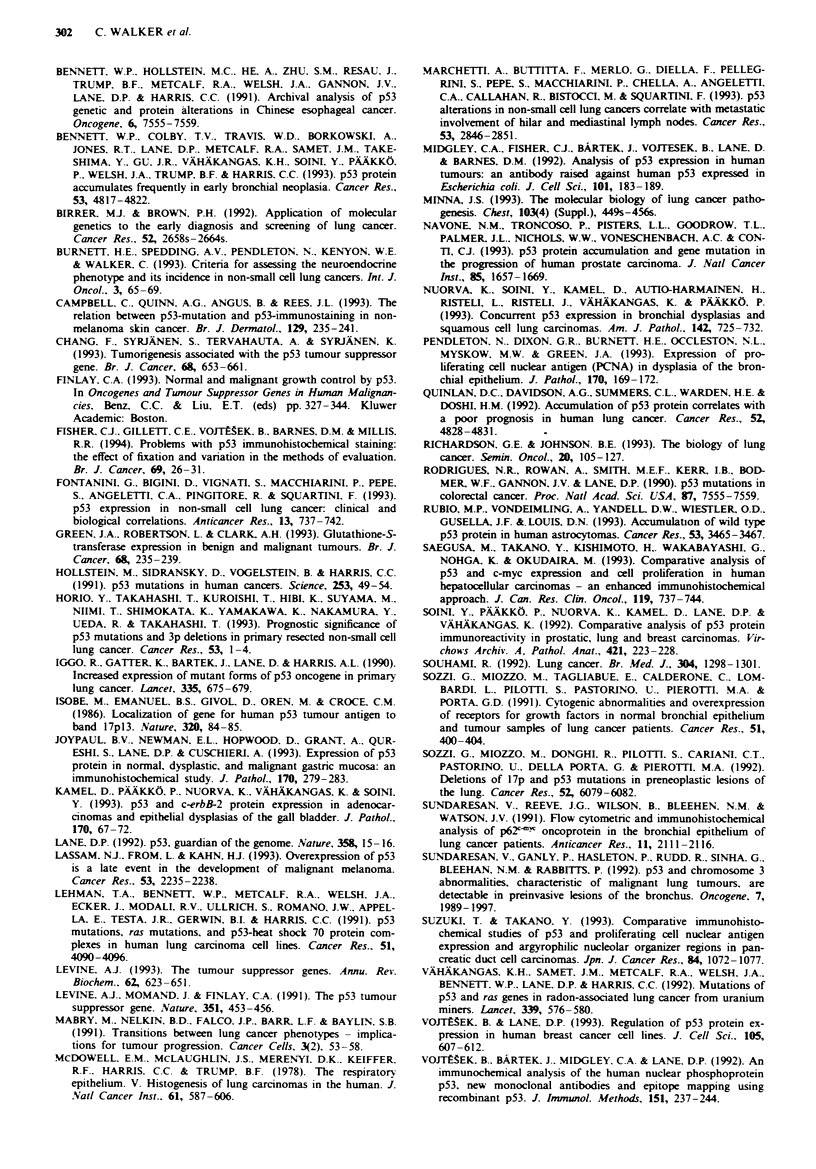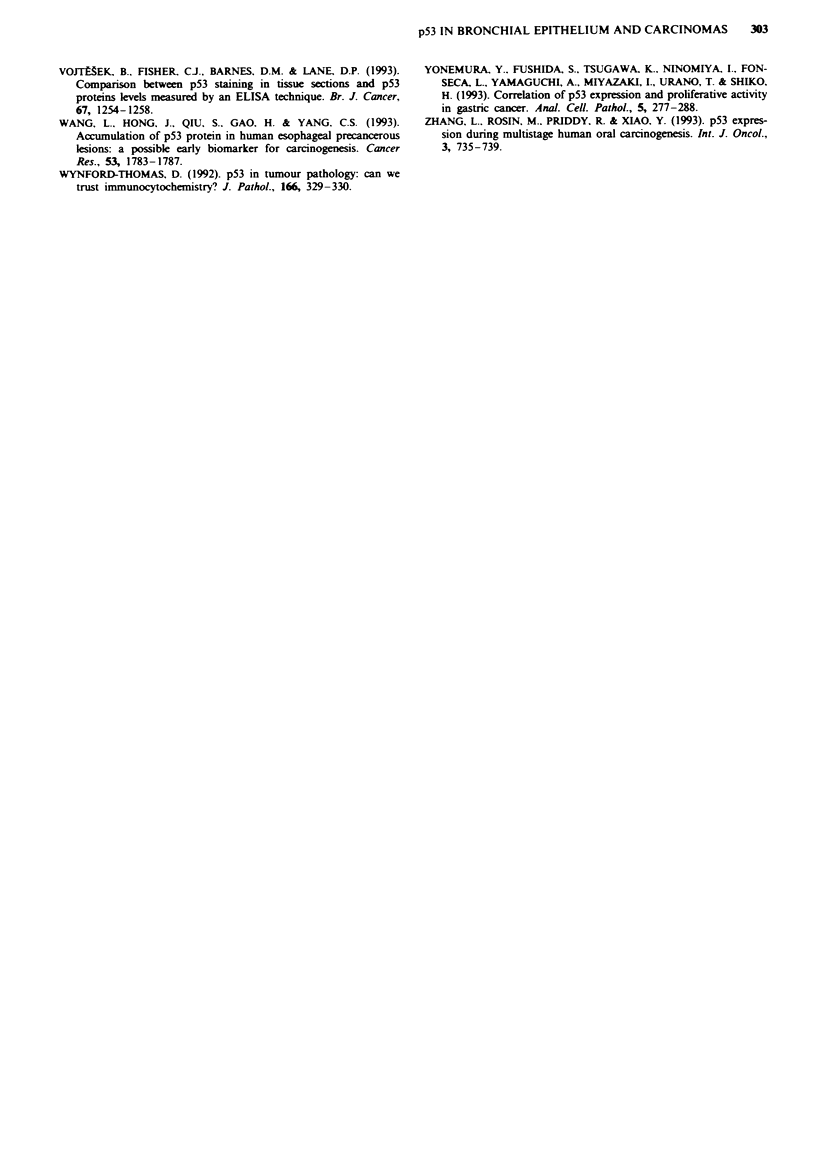# p53 expression in normal and dysplastic bronchial epithelium and in lung carcinomas.

**DOI:** 10.1038/bjc.1994.296

**Published:** 1994-08

**Authors:** C. Walker, L. J. Robertson, M. W. Myskow, N. Pendleton, G. R. Dixon

**Affiliations:** Clatterbridge Cancer Research Trust, J K Douglas Cancer Research Laboratory, Clatterbridge Hospital, Bebington, Wirral, UK.

## Abstract

**Images:**


Br. J. Cancer (1994), 70, 297-303 © Macmillan Press Ltd., 1994

### p53 expression in normal and dysplastic bronchial epithelium and in lung carcinomas

C. Walker[1], L.J. Robertson[1], M.W. Myskow[2], N. Pendleton[1] & G.R. Dixon[2]

[1]*Clatterbridge Cancer Research Trust, J K Douglas Cancer Research Laboratory, Clatterbridge Hospital, Bebington, Wirral L63 4JY, UK;* [2]*Department of Histopathology, Broadgreen Hospital, Liverpool L14 3LB, UK.*

**Summary** Bronchial epithelial dysplasia is thought to be a premalignant stage in the evolution of lung cancers. Using the CM-1 polyclonal antibody, we have examined the expression of the p53 protein in a larger series of bronchial dysplasias ($n = 60$) than hitherto investigated. The p53 protein was detected in 14% of mild, 25% of moderate and 59% of severe dysplasias; increased p53 expression correlated with the severity of dysplasia. p53-positive dysplasias had greater PCNA indices than p53-negative dysplasias. p53 expression in dysplastic tissues was compared with that in two groups of histologically normal epithelium: 14 bronchial biopsies from non-cancer patients of which all but one were negative and 32 bronchial margins from resected carcinomas, of which 17 showed infrequent solitary cells with p53-positive nuclei in predominantly basal locations scattered throughout the epithelium. These results for resection margins were confirmed by use of a second antibody, DO-1. Sixty-nine per cent of the corresponding carcinomas were p53 positive, but in 15 cases the p53 reactivity differed from resection margins. No correlation between p53 expression and any of the clinicopathological characteristics of these tumours was found. This study supports the observation that abnormal p53 expression may be an early but not obligatory event in malignant transformation in lung.

Lung cancer remains one of the most common cancers, for which therapies are currently inadequate and prognosis is often poor (Souhami, 1992; Richardson & Johnson, 1993). Improvements in the treatment of this disease would be greatly assisted by its early detection (Birrer & Brown, 1992).

It is now widely agreed that all lung cancers are derived from a common pluripotent stem cell capable of expressing a variety of phenotypes (Mabry *et al.*, 1991). Although the sequence of events in the histogenesis of lung cancer is unknown, bronchial epithelial dysplasia is thought to be a premalignant stage in the evolution of lung carcinomas (Auerbach *et al.*, 1962, 1979; McDowell *et al.*, 1978). Multistep genetic changes, which include activation of cellular proto-oncogenes and inactivation of tumour-suppressor genes, are associated with the development of human cancers and are thought to accompany the morphological changes that precede malignancy (Minna, 1993).

Currently the most commonly identified genetic change in human cancers is mutation in the p53 gene (Richardson & Johnson, 1993), located at position 13 on the short arm of chromosome 17 (Isobe *et al.*, 1986). This gene is a tumour-suppressor gene and encodes a 53 kDa nuclear phosphoprotein capable of binding to DNA and acting as a transcriptional factor (Finlay, 1993; Minna, 1993). The wild-type p53 protein inhibits cell proliferation, and loss of this activity leads to neoplastic transformation (Finlay, 1993; Levine, 1993). This protein has a short cellular half-life and is usually present in normal cells, under normal physiological conditions, in extremely small amounts, making it undetectable by standard immunohistochemical techniques (Iggo *et al.*, 1990; Rodrigues *et al.*, 1990; Chang *et al.*, 1993). Many mutations of the p53 gene, principally in exons 5-8 (Hollstein *et al.*, 1991), lead to a functional inactivation of the gene and a protein product unable to regulate transcription, ultimately resulting in deregulation of cell growth (Chang *et al.*, 1993; Finlay, 1993; Minna, 1993). Mutant p53 has an extended cellular half-life enabling immunohistochemical detection of the accumulated mutant protein in cell nuclei (Levine *et al.*, 1991). Although not all mutations lead to protein accumulation (Bennett *et al.*, 1991; Lehman *et al.*, 1991; Vähäkangas *et al.*, 1992), in many studies a correlation between the p53 protein detected immunocytochemically and p53 gene mutations has been found (Iggo *et al.*, 1990; Midgely *et al.*, 1992; Navone *et al.*, 1993). However, recent research has shown that not all immunohistochemically detected p53 results from mutation in the p53 gene (Lane, 1992; Wynford-Thomas, 1992; Chang *et al.*, 1993; Fisher *et al.*, 1994). p53 overexpression has been observed in many malignancies, including 60-70% of lung cancers (Iggo *et al.*, 1990; Soini *et al.*, 1992).

Investigation of p53 overexpression in premalignant tissues has led to the observations that alterations in the p53 gene arise as late events in the evolution of some cancers, e.g. in gastric carcinomas (Joypaul *et al.*, 1993), prostatic carcinomas (Navone *et al.*, 1993) or melanomas (Lassam *et al.*, 1993), whereas in others, e.g. oral (Zhang *et al.*, 1993), gall bladder (Kamel *et al.*, 1993) and oesophageal (Wang *et al.*, 1993) malignancies, abnormal p53 expression is an early event. In attempts to define the type and temporal sequence of somatic genetic changes that precede the onset of invasive lung cancer, recent studies have reported mutations and allelic deletions in the p53 gene in preinvasive bronchial lesions (Sozzi *et al.*, 1992; Sundaresen *et al.*, 1992). Immunodetectable p53 has been found in a few cases of bronchial dysplasia (Vähäkangas *et al.*, 1992; Sozzi *et al.*, 1992; Sundaresan *et al.*, 1992), and Nuorva *et al.* (1993) have reported that p53 overexpression correlated with the severity of dysplasia in 17 cases of dysplastic epithelium from cancer-bearing patients. Thus lesions in the p53 gene have been reported as possible early events in the development of lung cancers.

In this study we have investigated the immunohistochemical expression of the p53 protein in a series ($n = 60$) of bronchial epithelial dysplasias and related their positivity to severity of dysplasia and proliferating cell nuclear antigen (PCNA) indices (Pendleton *et al.*, 1993). p53 expression in dysplastic tissues was compared with that in histologically normal bronchial epithelium from non-lung cancer patients as well as from the resection margins of lung carcinomas. Expression of p53 in the corresponding tumours was also investigated.

### Materials and methods

*Lung tissues*

Sixty formalin-fixed, paraffin-embedded bronchial biopsies which had been reported to contain dysplastic epithelium were retrieved from the archives at the Histopathology

Correspondence: C. Walker
Received 5 January 1994; and in revised form 17 March 1994.

Department, Broadgreen Hospital, Liverpool. In 45 cases there was a concomitant diagnosis of lung cancer. Ten cases were either associated with a benign lesion or did not have any other form of pulmonary pathology. Clinical data were not available for the remaining five cases. Dysplasia was graded independently by two pathologists as mild, moderate or severe as described in Pendleton *et al.* (1993). Fourteen formalin-fixed, paraffin-embedded bronchial biopsies, taken from patients who did not have lung cancer at the time of biopsy and which contained epithelium reported as histologically normal, were also obtained from the files. Thirty-two formalin-fixed, paraffin-embedded specimens of lung carcinoma and their corresponding bronchial resection margins were collected prospectively by N. Pendleton from lobectomies or pneumonectomies performed at the Cardiothoracic Centre, Liverpool, UK. Patients received no other form of therapy either prior to or following surgery and were staged using UICC guidelines. Full clinical data were available for these cases.

### *Immunohistochemistry*

p53 immunoreactivity was determined using methods essentially similar to those described by Green *et al.* (1993). Five micron sections were cut, mounted on glass and dried overnight at 37°C, prior to dewaxing in xylene for 20 min and rehydration through alcohol. Endogenous peroxidase was destroyed by incubation in methanol containing 3% hydrogen peroxide (v/v) for 20 min. Sections were washed in water then immersed in Tris-buffered saline (TSB), pH 7.6, containing 0.1% bovine serum albumin (BSA) (Sigma cat. no. A-4503). This buffer was used for all subsequent washes and for dilution of antibodies. Sections were incubated with primary antibody for 30 min at 20°C, either the polyclonal antibody CM-1 (Novacastra) at 1:800 or the monoclonal antibody DO-1 (Ab-6) (Oncogene Science) at 1:100. These antibodies recognise both wild-type and mutant forms of p53 (Midgely *et al.*, 1992; Vojtesek *et al.*, 1992). After three washes in buffer, biotinylated swine anti-rabbit (Dako code no. E413) or biotinylated rabbit anti-mouse (Dako code no. E431) secondary antibodies were applied at dilutions of 1:300 and sections incubated for 30 min at 20°C. After three washes in buffer, staining was visualised by the avidin-biotin-peroxidase technique (Dako code no. 355), followed after three washes by incubation in phosphate-citrate buffer, pH 6.4, containing 0.05% 3',3-diaminobenzidine tetrahydrochloride and 0.3% hydrogen peroxide. After 5 min, the sections were washed in water, counterstained in haematoxylin, dehydrated and mounted in DPX mounting medium. Negative controls using normal swine serum for polyclonals and normal rabbit serum for monoclonals at 1:400 and TBS instead of the primary antibody were included in each staining run. Cos monkey cells transfected with the p53 oncogene were used as positive controls and were a gift from Dr J. Jenkins, Marie Cure Institute, UK.

Approximate percentages of nuclei that were p53 positive in normal or dysplastic epithelium and p53-positive tumour nuclei in carcinomas were assessed and scored as follows: 0, negative; 1, <10%; 2, 10-50%; 3, >50%. The intensity of staining in p53-positive nuclei was compared with the positive control and scored as follows: 0, negative; 1, clearly stained but with an intensity less than the positive control; 2, intensity equal to the positive control; 3, intensity greater than the positive control. The overall p53 score for each section was the sum of the distribution and the intensity scores. In some cases cytoplasmic staining was present; this was noted, but only nuclear staining was considered positive.

PCNA indices of dysplastic biopsies and resection margins had been determined previously (Pendleton *et al.*, 1993); in this study, these results were related to p53 reactivity.

### *Statistical analysis*

The significances of associations were determined using Fisher-Irwin's exact probability test. Mann-Whitney tests were used to compare PCNA indices for p53-positive and p53-negative groups. Spearman's rank correlation was used to compare severity of dysplasia with degree of p53 expression, and the Mann-Whitney test used to compare p53 expression between tissue groups. Survival data for p53-positive versus p53-negative tumours were analysed using the Peto log-rank test. Two-tailed probabilities are quoted for all statistical tests.

### Results

### *p53 positivity in normal and dysplastic bronchial epithelium*

p53 immunoreactivity was investigated using the CM-1 antibody in biopsies of normal and dysplastic bronchial epithelium. Twenty-eight of the 60 dysplastic biopsies were p53 positive compared with only one of the 14 normal bronchial biopsies taken from patients who did not have cancer at the time of biopsy (Table I).

In seven of the biopsies tumour was present in the same section as dysplastic epithelium; in five the p53 reactivity of tumour cells and dysplastic epithelium was in agreement, but in two cases dysplastic epithelium was p53 positive although adjacent tumour was negative.

### *p53 positivity and grade of dysplasia*

Of the biopsies investigated, dysplasia was mild in seven cases, moderate in 12 cases and severe in 41 cases. Using the CM-1 antibody, cells with clearly stained p53-positive nuclei were found in 14% of the mild, 25% of the moderate and 59% of the severe dysplasias (Figure 1 and Table II). In the one case of mild dysplasia found to be p53 positive, positive nuclei were present in <1% of the epithelial cells and were located basally and suprabasally scattered along the epithelium. In moderate and severe dysplasias, p53-positive cells were seen in basal and suprabasal layers, although in some cases these cells were present throughout the full thickness of the epithelium (Figure 1). p53-positive cells were frequently intensely stained and often present as foci of positive cells.

**Table I** p53 positivity in normal and dysplastic epithelium in bronchial biopsies

| *p53* | *Normal* | *Dysplasia* | *Total* |
|---|---|---|---|
| Negative | 13 | 32 | 45 |
| Positive | 1 | 28 | 29 |
| Total | 14 | 60 | 74 |

$P = 0.006$, Fisher-Irwin exact test (two-tailed).

**Figure 1** p53 immunoreactivity in severely dysplastic epithelium, showing nuclear staining. Scale bar = 20 μm.

p53 expression was found to correlate with the severity of dysplasia. p53 was detected significantly more often in severe dysplasias than in mild + moderate dysplasias or mild dysplasias (Table II). Significant differences between normal and dysplastic epithelium were only found when the severe dysplasias were included in the group analysed. Compared with normal epithelium p53 expression was significantly greater in all dysplasias (Table I), severe dysplasias and in moderate + severe dysplasias (Table II).

*p53 score and grade of dysplasia*

The system of p53 scoring used in these experiments (Table III) permitted a semiquantitative comparison of the degree of p53 expression in the various tissues examined. With increasing severity of dysplasia there was not only an increase in the percentage of cases demonstrating p53 staining but also an increase in the staining intensity of positive cells and an increase in the proportions of these positive cells. Thus, higher grades of dysplasia were associated with higher p53 scores; the Spearman rank correlation coefficient for the whole table (Table III) is 0.47 ($P = <0.0001$), and considering just the dysplasia cases it is 0.37 ($P = 0.002$). Comparison of p53 expression between the various grades of dysplasia by use of p53 score as in Table III results in more significant $P$-values by the Mann-Whitney test than obtained with the Fisher-Irwin tests of Table II.

*PCNA indices*

For 39 cases of bronchial dysplasia, PCNA indices had been determined previously (Pendleton *et al.*, 1993). p53-positive dysplasias had significantly greater PCNA indices than p53-negative dysplasias (Table IVa), indicating abnormal growth in these p53-positive biopsies.

*Bronchial carcinomas and resection margins*

In previous studies, a series of bronchial carcinomas (Burnett *et al.*, 1993) and their corresponding resection margins which contained histologically normal epithelium (Pendleton *et al.*, 1993) had been collected prospectively following surgery. Using the CM-1 antibody, p53-positive nuclei were seen in 22/32 (69%) of the tumours and in histologically normal epithelium in 17/32 (53%) of the resection margins (Table V). p53-positive cells in resection margins were predominantly basal, solitary and scattered throughout the epithelium (Figure 2a), and were less frequent than in tumour tissues (Figure 2b) or many samples of dysplastic epithelium. Many nuclei were weakly stained, but some showed a staining intensity similar to p53-positive tumour cells. Compared with dysplasias and tumour tissues the p53 scores of resection margins were low (Tables III and V), with only one case with a score of 3 and no higher scores. To confirm these results, sections of resection margins were stained with the monoclonal antibody DO-1; all of the cases positive for the CM-1 antibody were also DO-1 positive, but two cases (numbers 16 and 21) which were negative for CM-1 were clearly positive for DO-1.

Clearly stained p53-positive nuclei were significantly more often detected in histologically normal epithelium from the bronchial resection margins of lung cancer patients than in the normal bronchial epithelium from patients who did not have cancer ($P = 0.003$, Fisher-Irwin test). PCNA indices (Pendleton *et al.*, 1993) of p53-positive resection margins did not differ significantly from the p53-negative group (Table IVb).

The p53 scores for positive tumours were generally higher than the scores for normal or dysplastic epithelium (Tables III and V), indicating an increased level of p53 expression in tumours. p53-positive tumour nuclei were frequently intensely stained (Figure 2b) and often showed a patchy distribution within the tumour. In some cases p53 positivity was focal, and in some it was observed at the leading edge of the tumour. Because of the focal distribution of p53 positivity in

**Table II** p53 positivity and grade of dysplasia

| p53 | *Normal* | *Mild* | *Moderate* | *Severe* | *Total* |
|---|---|---|---|---|---|
| | *Histology* | *Dysplasia* | | | |
| Negative | 13 | 6 | 9 | 17 | 45 |
| Positive | 1 | 1 | 3 | 24 | 29 |
| Total | 14 | 7 | 12 | 41 | 74 |

Fisher-Irwin exact test (two-tailed probabilities): mild versus severe, 0.04; moderate versus severe, NS; mild + moderate versus severe, 0.01; mild versus normal, NS; moderate versus normal, NS; severe versus normal, 0.001; moderate + severe versus normal, 0.004; NS, not significant.

**Table III** p53 score

| p53 score | *Normal* | *Mild* | *Moderate* | *Severe* | *Total* |
|---|---|---|---|---|---|
| | *Histology* | *Dysplasia* | | | |
| 0 | 13 | 6 | 9 | 17 | 45 |
| 2 | 1 | 1 | 1 | 8 | 11 |
| 3-4 | 0 | 0 | 2 | 11 | 13 |
| 5-6 | 0 | 0 | 0 | 5 | 5 |
| Total | 14 | 7 | 12 | 41 | 74 |

$P$-values by Mann-Whitney test (two-tailed): mild versus severe, 0.03; moderate versus severe, 0.05; mild + moderate versus severe, 0.005.

**Table IV** PCNA indices

| | *Number of cases* | *PCNA index* *Median* | *Range* | P[a] |
|---|---|---|---|---|
| (a) *Dysplastic bronchial epithelium* | | | | |
| p53 negative | 22 | 10 | 0-88 | 0.0001 |
| p53 positive | 17 | 71 | 2-94 | |
| (b) *Normal epithelium in resection margins* | | | | |
| p53 negative | 15 | 5 | 0-15 | NS |
| p53 positive | 17 | 0 | 0-19 | |

[a]Two-tailed Mann-Whitney test. NS, not significant.

tumours, the need to examine multiple blocks has been reported (Soini *et al.*, 1992; Nuorva *et al.*, 1993). In this study, 20/32 of the tumours were p53 positive when one block from each case was examined. For every negative case, a further 2-4 blocks were investigated. This resulted in only two additional positive cases (tumour no. 10, for which two blocks were positive and one was negative, and tumour no. 21, for which two blocks were positive and two were negative).

Seven out of 11 (64%) adenocarcinomas, 10/15 (66%) squamous cell carcinomas, 1/1 small-cell-lung cancer (SCLC), 1/2 bronchial carcinoids, 1/1 large-cell carcinoma, 1/1 adenosquamous and 1/1 squamous/SCLC had immunodetectable p53. There was no significant difference in p53 expression in adenocarcinomas and squamous cell carcinomas. No correlation between p53 expression and tumour stage, TNM score or patient survival was found.

In 15 cases the p53 reactivity in the tumour differed from that found in the normal epithelium in corresponding resection margins; in ten cases the tumour was positive for p53 while the resection margins were negative; and in five cases the tumour was p53 negative although the normal epithelium contained p53-positive cells.

### Discussion

This study has investigated the immunohistochemical expression of the p53 protein in a larger single series of bronchial dysplasias than hitherto investigated (Sozzi *et al.*, 1992; Sun-

**Table V** Expression of p53 in bronchial carcinomas and resection margins

| | | *Carcinomas* | | | *Normal epithelium in resection margins* | | |
|---|---|---|---|---|---|---|---|
| *Case No.* | *Histology* | *p53 distribution score* | *p53 intensity score* | *p53 overall score* | *p53 distribution score* | *p53 intensity score* | *p53 overall score* |
| *State I* | | | | | | | |
| 1 | Adenocarcinoma | 3 | 3 | 6 | 1 | 1 | 2 |
| 2 | Adenocarcinoma | 1 | 1 | 2 | 0 | 0 | 0 |
| 3 | Squamous cell | 3 | 3 | 6 | 0 | 0 | 0 |
| 4 | Squamous cell | 3 | 3 | 6 | 0 | 0 | 0 |
| 5 | Squamous cell | 0 | 0 | 0 | 1 | 1 | 2 |
| 6 | Adenocarcinoma | 0 | 0 | 0 | 1 | 1 | 2 |
| 7 | Carcinoid | 1 | 1 | 2 | 1 | 1 | 2 |
| *State II* | | | | | | | |
| 8 | Adenocarcinoma | 2 | 2 | 4 | 1 | 1 | 2 |
| 9 | Squamous cell | 2 | 1 | 3 | 1 | 1 | 2 |
| 10 | Squamous cell | 2 | 2 | 4 | 1 | 2 | 3 |
| 11 | Large cell | 3 | 2 | 5 | 0 | 0 | 0 |
| 12 | Adenocarcinoma | 0 | 0 | 0 | 0 | 0 | 0 |
| 13 | Carcinoid | 0 | 0 | 0 | 0 | 0 | 0 |
| 14 | Squamous cell | 2 | 2 | 4 | 0 | 0 | 0 |
| 15 | Squamous cell | 3 | 2 | 5 | 1 | 1 | 2 |
| 16 | Squamous cell | 3 | 2 | 5 | 0 | 0 | 0 |
| *Stage IIIa* | | | | | | | |
| 17 | Adenocarcinoma | 3 | 2 | 5 | 1 | 1 | 2 |
| 18 | Squamous cell | 3 | 2 | 5 | 1 | 1 | 2 |
| 19 | Adeno/squamous | 2 | 2 | 4 | 0 | 0 | 0 |
| 20 | Squamous cell | 3 | 2 | 5 | 0 | 0 | 0 |
| 21 | Adenocarcinoma | 1 | 1 | 2 | 0 | 0 | 0 |
| 22 | Adenocarcinoma | 1 | 1 | 2 | 1 | 1 | 2 |
| 23 | Small cell | 3 | 2 | 5 | 1 | 1 | 2 |
| 24 | Squamous cell | 0 | 0 | 0 | 1 | 1 | 2 |
| 25 | Squamous cell | 0 | 0 | 0 | 1 | 1 | 2 |
| 26 | Adenocarcinoma | 1 | 2 | 3 | 1 | 1 | 2 |
| 27 | Squamous/small cell | 3 | 2 | 5 | 0 | 0 | 0 |
| 28 | Adenocarcinoma | 0 | 0 | 0 | 1 | 1 | 2 |
| 29 | Adenocarcinoma | 0 | 0 | 0 | 0 | 0 | 0 |
| 30 | Squamous cell | 0 | 0 | 0 | 0 | 0 | 0 |
| 31 | Squamous cell | 0 | 0 | 0 | 0 | 0 | 0 |
| 32 | Squamous cell | 3 | 3 | 6 | 1 | 1 | 2 |

daresan *et al.*, 1992; Vähäkangas *et al.*, 1992; Nuorva *et al.*, 1993) and supports the conclusion that p53 overexpression correlates significantly with severity of bronchial dysplasia. Detection of the p53 protein in mild and moderate dysplasias suggests that abnormal p53 expression is an early event in the malignant transformation process in lung; these results support the observations that somatic genetic changes in the p53 gene occur in preinvasive lesions of the lung (Sundaresan *et al.*, 1992; Vähäkangas *et al.*, 1992).

During the course of the preparation of this manuscript, Bennett *et al.* (1993), also using the CM-1 antibody in a series of 34 cases, have reported that the p53 protein accumulates frequently in early bronchial neoplasia. Our study supports their conclusions but differs in that biopsies, not resected tumours, were examined and all tissues were derived from a single treatment centre. Combining the results of our study with those of other published studies (Sozzi *et al.*, 1992; Sundaresan *et al.*, 1992; Vähäkangas *et al.*, 1992; Bennett *et al.*, 1993; Nuorva *et al.*, 1993), p53 expression has so far been investigated in a combined total of 23 mild, 31 moderate and 77 severe dysplasias. Provided that assessment of positivity is similar in all studies, 19% of the mild, 28% of the moderate and 63% of the severe dysplasias have been found to be p53 positive.

In other similar studies investigating the expression of the p53 protein in premalignant lesions of lung (Bennett *et al.*, 1993; Nuorva *et al.*, 1993) and other tissues (Joypaul *et al.*, 1993; Kamel *et al.*, 1993), results were analysed by assessment of p53 positivity. In this study, analysis was either by comparison of p53-positive and -negative groups or by use of a p53 scoring system similar to that described by Vojtěšek *et al.* (1993). The advantage of this scoring system is that it allows comparison of the degree of p53 expression between tissue groups. The p53-positive group, equivalent to the positive group in other similar studies, had a p53 score of two or more. Cells with the p53 score for intensity of 1 were clearly p53 positive and were found in tumours as well as in dysplasias and normal tissues.

Unlike other studies of preinvasive lung lesions (Sozzi *et al.*, 1992; Sundaresan *et al.*, 1992; Vähäkangas *et al.*, 1992; Bennett *et al.*, 1993; Nuorva *et al.*, 1993), PCNA indices for many of the dysplasias in this series had been determined (Pendleton *et al.*, 1993). The greater PCNA indices of the p53-positive group indicates that p53-positive dysplasias contain higher proportions of cells in the proliferative phase of the cell cycle; this suggests that p53-positive dysplasias may have abnormalities in their growth control mechanisms. It is possible that alterations in the p53 gene confer a growth advantage on these cells, leading to expansion of p53-positive cells as severity of dysplasia increases. A close relation between p53 overexpression and PCNA indices has also been observed in pancreatic duct cell carcinomas (Suzuki & Takano, 1993), hepatocellular carcinomas (Saegusa *et al.*, 1993) and gastric cancers (Yonemura *et al.*, 1993).

In this study, p53 expression in dysplastic tissues was compared with two groups of histologically normal epithelium. All but one of the first group, taken from patients who did not have cancer at the time of biopsy, were negative. Comparison of p53 expression in this group with that in dysplastic bronchial biopsies showed a highly significant difference between these groups. The second group of histologically normal epithelium analysed, from the resection margins of bronchial carcinomas, showed p53-positive cells in a high proportion of cases, indicating differences in the normal bronchial epithelium of cancer and non-cancer patients. The patient from whom the one p53-positive biopsy of normal bronchial epithelium was obtained did not develop lung cancer in 9 months following biopsy.

**Figure 2** p53 immunoreactivity (**a**) in a resection margin from a squamous cell carcinoma showing nuclear staining in a few cells scattered along the epithelium (scale bar = 10 μm), and (**b**) in a squamous cell carcinoma showing strong nuclear staining in a high proportion of tumour cells (scale bar = 20 μm).

The intensity and distribution of p53-positive cells in histologically normal epithelium was similar to that observed in the one case of mild dysplasia found to be p53 positive. In all but one case, the intensity of p53-positive nuclei was scored as 1. These results reflect genuine p53 overexpression in these cells and are not experimental artefact because: (a) a second antibody showed similar results, (b) a high proportion of resection margins contained p53-positive cells and (c) these cells, although often weakly stained and infrequently distributed, were clearly evident against a background of p53-negative epithelial and mesenchymal tissues. Similar p53 positivity was found in 3/6 histologically normal cells in oesophageal epithelium (Wang *et al.*, 1993), and Vähäkangas *et al.* (1992) commented that occasional p53-positive normal-appearing bronchial mucosal cells were observed in the normal tissues adjacent to a lung tumour from a uranium miner. Bennett *et al.* (1993) have reported that all 22 examples of normal mucosa examined in their series from bronchial resections were p53 negative, although four cases were reported to have 'equivocal' stain.

It has recently been observed that not all immunodetectable p53 reacts with antibodies specific for the mutant form (Fontanini *et al.*, 1993; Rubio *et al.*, 1993) or is associated with mutation in the p53 gene (Vähäkangas *et al.*, 1992; Campbell *et al.*, 1993; Marchetti *et al.*, 1993; Rubio *et al.*, 1993; Vojtěšek & Lane, 1993). Elevated amounts of wild-type p53 are found in cells in a variety of circumstances, e.g. in cells undergoing DNA repair, in which accumulation of the p53 protein mediates arrest in the $G_1$ phase of the cell cycle, or in association with abnormal levels of viral or cellular proteins known to bind to the p53 protein, such as the SV40 large T antigen or the *MDM2* gene product (Lane, 1992; Bennett *et al.*, 1993; Chang *et al.*, 1993; Levine, 1993). Thus p53 overexpression detected by immunocytochemistry may reflect not gene mutation but some post-translational mechanism.

p53 positivity in the normal mucosa of resection margins did not result in a measurable increase in proliferation, as indicated by PCNA indices. This may suggest that the mechanism whereby the p53 protein is elevated in normal mucosa differs from that in dysplasia. Whatever the mechanism to account for these p53-positive cells in normal bronchial mucosa, it seems that their presence, even if not associated with mutation in the p53 gene, indicates abnormalities that are not reflected in the histological appearance of these cells. Cytogenetic abnormalities and overexpression of p62-*myc*, epidermal growth factor receptor (EGFR) and HER-2/*neu* have also been observed in histologically normal epithelium from the resection margins of lung cancer patients (Sozzi *et al.*, 1991; Sundaresan *et al.*, 1991), suggesting that cytogenetic instability or misregulation of normal growth controls precedes morphological change and may be early events in the transition from normal epithelium to invasive cancer. Such changes may contribute to the mechanism whereby lung cancer patients have an increased tendency towards the formation of a second primary lung cancer (Sozzi *et al.*, 1991). p53 abnormality in normal and dysplastic tissues was not necessarily associated with p53 overexpression in tumours. Such results suggest that early lesions in the p53 gene are only one of a number of such genetic alterations which, following further multiple and complex genetic changes, lead to the formation of a carcinoma.

The number of p53-positive tumours in the series (69% overall and 68% for non-small-cell lung cancers) agreed well with the incidence of p53 positivity for lung cancers reported in some studies (Iggo *et al.*, 1990; Fontanini *et al.*, 1993; Marchetti *et al.*, 1993) but was higher than that found in others (Quinlan *et al.*, 1992; Soini *et al.*, 1992). Although the number of tumours in this series was small, no correlation in p53 overexpression was found with any of the clinical characteristics of these tumours. This contrasts with reports of a relationship between p53 overexpression and poor prognosis and shortened survival (Quinlan *et al.*, 1992; Horio *et al.*, 1993), tumour grade (Soini *et al.*, 1992) or lymph node involvement (Fontanini *et al.*, 1993; Marchetti *et al.*, 1993) and a greater incidence in squamous cell carcinomas compared with other types of lung carcinoma (Iggo *et al.*, 1990; Soini *et al.*, 1992). Further investigation of a larger series of tumours from this geographical region would be necessary to relate p53 positivity to clinicopathological features of the disease as presented in Merseyside.

This study supports the observation that abnormal p53 expression is an early but not obligatory event in the evolution of lung cancers. Immunodetection of p53 overexpression in bronchial epithelium may be a useful tool in the identification of those early lesions which may progress to malignancy.

This work was supported by Clatterbridge Cancer Research Trust. The authors would like to thank Dr M.R. Daniel for helpful discussion and Dr I.R. Campbell for assistance with statistical analyses.

### References

AUERBACH, O., STOUT, A.P., HAMMOND, E.C. & GARFINKEL, L. (1962). Changes in bronchial epitheium in relation to sex, age, residence, smoking and pneumonia. *N. Engl. J. Med.*, **267**, 111-125.

AUERBACH, O., HAMMOND, E.C. & GARFINKEL, L. (1979). Changes in bronchial epithelium in relation to cigarette smoking, 1955-1960 vs 1930-1977. *New Engl. J. Med.*, **300**, 381-385.

BENNETT, W.P., HOLLSTEIN, M.C., HE, A., ZHU, S.M., RESAU, J., TRUMP, B.F., METCALF, R.A., WELSH, J.A., GANNON, J.V., LANE, D.P. & HARRIS, C.C. (1991). Archival analysis of p53 genetic and protein alterations in Chinese esophageal cancer. *Oncogene*, **6**, 7555-7559.

BENNETT, W.P., COLBY, T.V., TRAVIS, W.D., BORKOWSKI, A., JONES, R.T., LANE, D.P., METCALF, R.A., SAMET, J.M., TAKESHIMA, Y., GU, J.R., VÄHÄKANGAS, K.H., SOINI, Y., PÄÄKKÖ, P., WELSH, J.A., TRUMP, B.F. & HARRIS, C.C. (1993). p53 protein accumulates frequently in early bronchial neoplasia. *Cancer Res.*, **53**, 4817-4822.

BIRRER, M.J. & BROWN, P.H. (1992). Application of molecular genetics to the early diagnosis and screening of lung cancer. *Cancer Res.*, **52**, 2658s-2664s.

BURNETT, H.E., SPEDDING, A.V., PENDLETON, N., KENYON, W.E. & WALKER, C. (1993). Criteria for assessing the neuroendocrine phenotype and its incidence in non-small cell lung cancers. *Int. J. Oncol.*, **3**, 65-69.

CAMPBELL, C., QUINN, A.G., ANGUS, B. & REES, J.L. (1993). The relation between p53-mutation and p53-immunostaining in non-melanoma skin cancer. *Br. J. Dermatol.*, **129**, 235-241.

CHANG, F., SYRJÄNEN, S., TERVAHAUTA, A. & SYRJÄNEN, K. (1993). Tumorigenesis associated with the p53 tumour suppressor gene. *Br. J. Cancer*, **68**, 653-661.

FINLAY, C.A. (1993). Normal and malignant growth control by p53. In *Oncogenes and Tumour Suppressor Genes in Human Malignancies*, Benz, C.C. & Liu, E.T. (eds) pp. 327-344. Kluwer Academic: Boston.

FISHER, C.J., GILLETT, C.E., VOJTĚŠEK, B., BARNES, D.M. & MILLIS, R.R. (1994). Problems with p53 immunohistochemical staining: the effect of fixation and variation in the methods of evaluation. *Br. J. Cancer*, **69**, 26-31.

FONTANINI, G., BIGINI, D., VIGNATI, S., MACCHIARINI, P., PEPE, S., ANGELETTI, C.A., PINGITORE, R. & SQUARTINI, F. (1993). p53 expression in non-small cell lung cancer: clinical and biological correlations. *Anticancer Res.*, **13**, 737-742.

GREEN, J.A., ROBERTSON, L. & CLARK, A.H. (1993). Glutathione-*S*-transferase expression in benign and malignant tumours. *Br. J. Cancer*, **68**, 235-239.

HOLLSTEIN, M., SIDRANSKY, D., VOGELSTEIN, B. & HARRIS, C.C. (1991). p53 mutations in human cancers. *Science*, **253**, 49-54.

HORIO, Y., TAKAHASHI, T., KUROISHI, T., HIBI, K., SUYAMA, M., NIIMI, T., SHIMOKATA, K., YAMAKAWA, K., NAKAMURA, Y., UEDA, R. & TAKAHASHI, T. (1993). Prognostic significance of p53 mutations and 3p deletions in primary resected non-small cell lung cancer. *Cancer Res.*, **53**, 1-4.

IGGO, R., GATTER, K., BARTEK, J., LANE, D. & HARRIS, A.L. (1990). Increased expression of mutant forms of p53 oncogene in primary lung cancer. *Lancet*, **335**, 675-679.

ISOBE, M., EMANUEL, B.S., GIVOL, D., OREN, M. & CROCE, C.M. (1986). Localization of gene for human p53 tumour antigen to band 17p13. *Nature*, **320**, 84-85.

JOYPAUL, B.V., NEWMAN, E.L., HOPWOOD, D., GRANT, A., QURESHI, S., LANE, D.P. & CUSCHIERI, A. (1993). Expression of p53 protein in normal, dysplastic, and malignant gastric mucosa: an immunohistochemical study. *J. Pathol.*, **170**, 279-283.

KAMEL, D., PÄÄKKÖ, P., NUORVA, K., VÄHÄKANGAS, K. & SOINI, Y. (1993). p53 and c-*erbB*-2 protein expression in adenocarcinomas and epithelial dysplasias of the gall bladder. *J. Pathol.*, **170**, 67-72.

LANE, D.P. (1992). p53, guardian of the genome. *Nature*, **358**, 15-16.

LASSAM, N.J., FROM, L. & KAHN, H.J. (1993). Overexpression of p53 is a late event in the development of malignant melanoma. *Cancer Res.*, **53**, 2235-2238.

LEHMAN, T.A., BENNETT, W.P., METCALF, R.A., WELSH, J.A., ECKER, J., MODALI, R.V., ULLRICH, S., ROMANO, J.W., APPELLA, E., TESTA, J.R., GERWIN, B.I. & HARRIS, C.C. (1991). p53 mutations, *ras* mutations, and p53-heat shock 70 protein complexes in human lung carcinoma cell lines. *Cancer Res.*, **51**, 4090-4096.

LEVINE, A.J. (1993). The tumour suppressor genes. *Annu. Rev. Biochem.*, **62**, 623-651.

LEVINE, A.J., MOMAND, J. & FINLAY, C.A. (1991). The p53 tumour suppressor gene. *Nature*, **351**, 453-456.

MABRY, M., NELKIN, B.D., FALCO, J.P., BARR, L.F. & BAYLIN, S.B. (1991). Transitions between lung cancer phenotypes - implications for tumour progression. *Cancer Cells*, **3**(2), 53-58.

McDOWELL, E.M., McLAUGHLIN, J.S., MERENYI, D.K., KEIFFER, R.F., HARRIS, C.C. & TRUMP, B.F. (1978). The respiratory epithelium. V. Histogenesis of lung carcinomas in the human. *J. Natl Cancer Inst.*, **61**, 587-606.

MARCHETTI, A., BUTTITTA, F., MERLO, G., DIELLA, F., PELLEGRINI, S., PEPE, S., MACCHIARINI, P., CHELLA, A., ANGELETTI, C.A., CALLAHAN, R., BISTOCCI, M. & SQUARTINI, F. (1993). p53 alterations in non-small cell lung cancers correlate with metastatic involvement of hilar and mediastinal lymph nodes. *Cancer Res.*, **53**, 2846-2851.

MIDGLEY, C.A., FISHER, C.J., BÁRTEK, J., VOJTESEK, B., LANE, D. & BARNES, D.M. (1992). Analysis of p53 expression in human tumours: an antibody raised against human p53 expressed in *Escherichia coli*. *J. Cell Sci.*, **101**, 183-189.

MINNA, J.S. (1993). The molecular biology of lung cancer pathogenesis. *Chest*, **103**(4) (Suppl.), 449s-456s.

NAVONE, N.M., TRONCOSO, P., PISTERS, L.L., GOODROW, T.L., PALMER, J.L., NICHOLS, W.W., VONESCHENBACH, A.C. & CONTI, C.J. (1993). p53 protein accumulation and gene mutation in the progression of human prostate carcinoma. *J. Natl Cancer Inst.*, **85**, 1657-1669.

NUORVA, K., SOINI, Y., KAMEL, D., AUTIO-HARMAINEN, H., RISTELI, L., RISTELI, J., VÄHÄKANGAS, K. & PÄÄKKÖ, P. (1993). Concurrent p53 expression in bronchial dysplasias and squamous cell lung carcinomas. *Am. J. Pathol.*, **142**, 725-732.

PENDLETON, N., DIXON, G.R., BURNETT, H.E., OCCLESTON, N.L., MYSKOW, M.W. & GREEN, J.A. (1993). Expression of proliferating cell nuclear antigen (PCNA) in dysplasia of the bronchial epithelium. *J. Pathol.*, **170**, 169-172.

QUINLAN, D.C., DAVIDSON, A.G., SUMMERS, C.L., WARDEN, H.E. & DOSHI, H.M. (1992). Accumulation of p53 protein correlates with a poor prognosis in human lung cancer. *Cancer Res.*, **52**, 4828-4831.

RICHARDSON, G.E. & JOHNSON, B.E. (1993). The biology of lung cancer. *Semin. Oncol.*, **20**, 105-127.

RODRIGUES, N.R., ROWAN, A., SMITH, M.E.F., KERR, I.B., BODMER, W.F., GANNON, J.V. & LANE, D.P. (1990). p53 mutations in colorectal cancer. *Proc. Natl Acad. Sci. USA*, **87**, 7555-7559.

RUBIO, M.P., VONDEIMLING, A., YANDELL, D.W., WIESTLER, O.D., GUSELLA, J.F. & LOUIS, D.N. (1993). Accumulation of wild type p53 protein in human astrocytomas. *Cancer Res.*, **53**, 3465-3467.

SAEGUSA, M., TAKANO, Y., KISHIMOTO, H., WAKABAYASHI, G., NOHGA, K. & OKUDAIRA, M. (1993). Comparative analysis of p53 and c-myc expression and cell proliferation in human hepatocellular carcinomas - an enhanced immunohistochemical approach. *J. Can. Res. Clin. Oncol.*, **119**, 737-744.

SOINI, Y., PÄÄKKÖ, P., NUORVA, K., KAMEL, D., LANE, D.P. & VÄHÄKANGAS, K. (1992). Comparative analysis of p53 protein immunoreactivity in prostatic, lung and breast carcinomas. *Virchows Archiv. A, Pathol. Anat.*, **421**, 223-228.

SOUHAMI, R. (1992). Lung cancer. *Br. Med. J.*, **304**, 1298-1301.

SOZZI, G., MIOZZO, M., TAGLIABUE, E., CALDERONE, C., LOMBARDI, L., PILOTTI, S., PASTORINO, U., PIEROTTI, M.A. & PORTA, G.D. (1991). Cytogenic abnormalities and overexpression of receptors for growth factors in normal bronchial epithelium and tumour samples of lung cancer patients. *Cancer Res.*, **51**, 400-404.

SOZZI, G., MIOZZO, M., DONGHI, R., PILOTTI, S., CARIANI, C.T., PASTORINO, U., DELLA PORTA, G. & PIEROTTI, M.A. (1992). Deletions of 17p and p53 mutations in preneoplastic lesions of the lung. *Cancer Res.*, **52**, 6079-6082.

SUNDARESAN, V., REEVE, J.G., WILSON, B., BLEEHEN, N.M. & WATSON, J.V. (1991). Flow cytometric and immunohistochemical analysis of $p62^{c\text{-}myc}$ oncoprotein in the bronchial epithelium of lung cancer patients. *Anticancer Res.*, **11**, 2111-2116.

SUNDARESAN, V., GANLY, P., HASLETON, P., RUDD, R., SINHA, G., BLEEHAN, N.M. & RABBITTS, P. (1992). p53 and chromosome 3 abnormalities, characteristic of malignant lung tumours, are detectable in preinvasive lesions of the bronchus. *Oncogene*, **7**, 1989-1997.

SUZUKI, T. & TAKANO, Y. (1993). Comparative immunohistochemical studies of p53 and proliferating cell nuclear antigen expression and argyrophilic nucleolar organizer regions in pancreatic duct cell carcinomas. *Jpn. J. Cancer Res.*, **84**, 1072-1077.

VÄHÄKANGAS, K.H., SAMET, J.M., METCALF, R.A., WELSH, J.A., BENNETT, W.P., LANE, D.P. & HARRIS, C.C. (1992). Mutations of p53 and *ras* genes in radon-associated lung cancer from uranium miners. *Lancet*, **339**, 576-580.

VOJTĚŠEK, B. & LANE, D.P. (1993). Regulation of p53 protein expression in human breast cancer cell lines. *J. Cell Sci.*, **105**, 607-612.

VOJTĚŠEK, B., BÁRTEK, J., MIDGLEY, C.A. & LANE, D.P. (1992). An immunochemical analysis of the human nuclear phosphoprotein p53, new monoclonal antibodies and epitope mapping using recombinant p53. *J. Immunol. Methods*, **151**, 237-244.

VOJTĚŠEK, B., FISHER, C.J., BARNES, D.M. & LANE, D.P. (1993). Comparison between p53 staining in tissue sections and p53 proteins levels measured by an ELISA technique. *Br. J. Cancer*, **67**, 1254-1258.

WANG, L., HONG, J., QIU, S., GAO, H. & YANG, C.S. (1993). Accumulation of p53 protein in human esophageal precancerous lesions: a possible early biomarker for carcinogenesis. *Cancer Res.*, **53**, 1783-1787.

WYNFORD-THOMAS, D. (1992). p53 in tumour pathology: can we trust immunocytochemistry? *J. Pathol.*, **166**, 329-330.

YONEMURA, Y., FUSHIDA, S., TSUGAWA, K., NINOMIYA, I., FONSECA, L., YAMAGUCHI, A., MIYAZAKI, I., URANO, T. & SHIKO, H. (1993). Correlation of p53 expression and proliferative activity in gastric cancer. *Anal. Cell. Pathol.*, **5**, 277-288.

ZHANG, L., ROSIN, M., PRIDDY, R. & XIAO, Y. (1993). p53 expression during multistage human oral carcinogenesis. *Int. J. Oncol.*, **3**, 735-739.